# Hydrothermal Synthesis and Photocatalytic Property of β-Ga_2_O_3_ Nanorods

**DOI:** 10.1186/s11671-015-1070-5

**Published:** 2015-09-16

**Authors:** L. Sivananda Reddy, Yeong Hwan Ko, Jae Su Yu

**Affiliations:** Department of Electronics and Radio Engineering, Institute for Wearable Convergence Electronics, Kyung Hee University, 1 Seocheon-dong, Giheung-gu, Yongin-si, Gyeonggi-do 446-701 Republic of Korea

**Keywords:** Gallium oxides, Nanostructures, Chemical synthesis, Photocatalytic properties

## Abstract

Gallium oxide (Ga_2_O_3_) nanorods were facilely prepared by a simple hydrothermal synthesis, and their morphology and photocatalytic property were studied. The gallium oxide hydroxide (GaOOH) nanorods were formed in aqueous growth solution containing gallium nitrate and ammonium hydroxide at 95 °C of growth temperature. Through the calcination treatment at 500 and 1000 °C for 3 h, the GaOOH nanorods were converted into single crystalline α-Ga_2_O_3_ and β-Ga_2_O_3_ phases. From X-ray diffraction analysis, it could be confirmed that a high crystalline quality of β-Ga_2_O_3_ nanorods was achieved by calcinating at 1000 °C. The thermal behavior of the Ga_2_O_3_ nanorods was also investigated by differential thermal analysis, and their vibrational bands were identified by Fourier transform infrared spectroscopy. In order to examine the photocatalytic activity of samples, the photodegradation of Rhodamine B solution was observed under UV light irradiation. As a result, the α-Ga_2_O_3_ and β-Ga_2_O_3_ nanorods exhibited high photodegeneration efficiencies of 62 and 79 %, respectively, for 180 min of UV irradiation time.

## Background

In recent years, various fabrication methods of photocatalytic products have been developed for the photodegradation of organic and inorganic pollutants in the environment [1, 2]. Particularly, inorganic semiconductor nanomaterials have been considered to be promising for photocatalyst applications because they provide good physical and chemical properties with large surface area, and a variety of morphologies, such as nanorods, cubes, spheres, and flowers, could be achieved by the chemical synthesis [3–8]. In addition to the morphology and surface area of particles, the other factors which can influence the catalytic activity are pore volume, pore size, crystallinity, defect sites, exposed facets, etc. The electron transport mechanism and the exposed facets are related to the morphology of particles. In the one-dimensional (1D) morphology, the generation of electron charge carriers is higher along the elongated nanostructures and gives rise to fast transport of charge carriers, due to the hampering of recombination of charge carriers. Hence, 1D nanostructures are gaining more importance for their use in different applications as seen by latest reports [9, 10]. For example, Liu et al. studied the morphology-dependent photocatalytic properties of bare zinc oxide nanocrystals [11], which indicated that the rod-shaped ZnO nanostructures have higher photocatalytic activity than the multi-layer disks or truncated hexagonal cones. Similarly, Han et al. also studied the morphology-related properties of nano/microstructured ZnO crystallites [12]. Gallium oxide (Ga_2_O_3_) nanostructures have been recognized as an important material for several applications including catalysts, gas sensors, solar cells, and photodetectors due to their wide bandgap energy (*E*_g_ = 4.2 to 4.7 eV) and good luminescence properties [13–16]. Typically, the Ga_2_O_3_ nanostructures could be obtained by calcination of gallium oxide hydroxide (GaOOH) which has been synthesized via various fabrication routes including thermal evaporation, hydrothermal, sol-gel, and microwave-assisted methods [17–19].

In order to obtain such GaOOH nanostructures by using a facile hydrothermal method, gallium nitrate was chemically reacted with various alkalis such as NaOH, NH_4_OH, KOH, and Na_2_CO_3_ [20, 21]. Then, the phase of the GaOOH nanostructures was changed into the rhombohedral crystal structure (α-Ga_2_O_3_) and monoclinic crystal structure (β-Ga_2_O_3_) after calcination [22, 23]. The crystal structures of Ga_2_O_3_ nanostructures strongly affect the chemical property and photocatalytic activity, but they still have not been completely studied. In this work, we prepared and characterized two kinds of Ga_2_O_3_ nanorods (α- and β-crystal structures) via a facile hydrothermal synthesis and proper calcination. By changing the temperature of the growth solution, the morphological properties of GaOOH nanostructures were investigated. Previously, there are so many reports on the preparation of Ga_2_O_3_ nanorods by hydrothermal and solvothermal methods which include complex instruments, making it an expensive approach. Girija et al. synthesized Ga_2_O_3_ nanostructures by the reflux method [24], Li et al. and Wang et al. used hydrothermal synthesis (autoclave) [25, 26], and Zhang et al. used the solvothermal method (autoclave) to obtain the Ga_2_O_3_ nanostructures [27]. However, in this work, we chose a simple and cost-effective process in which a beaker on a hotplate is used to produce crystalline Ga_2_O_3_ nanostructures. To characterize the structural property and functional group of the prepared samples, field-emission scanning electron microscopy (FE-SEM), X-ray diffraction (XRD), transmission electron microscopy (TEM), and Fourier transform infrared spectroscopy (FT-IR) analyses were employed. Also, the photocatalytic feasibility of Ga_2_O_3_ nanorods was evaluated by measuring the UV-vis absorption spectrum with a Rhodamine B (RhB) solution for environmental applications.

## Methods

The GaOOH nanorods were synthesized by the hydrothermal synthesis method using analytical pure grade chemicals. To make the growth solution, 0.1 M of gallium(III) nitrate hydrate (Ga(NO_3_)_3_·nH_2_O) was dissolved in 100 ml of de-ionized (DI) water. Then, the aqueous solution was heated on a hot plate at different temperatures from room temperature to 95 °C. While the temperature of the growth solution was maintained, ammonium hydroxide (NH_4_OH) was slowly added into the solution until a pH of 9 is reached. The final solution was then heated for 5 h to get a white precipitate of GaOOH nanorods. After the solution was naturally cooled down to room temperature, the precipitate was filtered and washed with DI water. Then, the sample was dried in an oven at 70 °C for 5 h under ambient atmosphere. Further, the as-prepared GaOOH nanorods were calcinated at different temperatures of 500, 800, and 1000 °C for 3 h to obtain the α- and β-Ga_2_O_3_ powders.

For the morphological and structural analysis, FE-SEM (LEO SUPRA 55, Carl Zeiss), TEM (JEM-2100F, JEOL), and XRD (M18XHF-SRA, Mac Science) measurements were utilized. The thermal behavior of the GaOOH nanorods was investigated by thermogravimetric analysis-differential scanning calorimetry (TGA-DSC: SDT Q600 V8.3 Build 101). The FT-IR spectrum was scanned and analyzed in the wavenumber range of 4000–400 cm^−1^ by using a FT-IR measurement system (Spectrum 100, PerkinElmer). The photocatalytic properties of α- and β-Ga_2_O_3_ nanorods for the degradation of Rhodamine B (RhB) aqueous solution were characterized by measuring the absorbance of the irradiated solution. For UV irradiation, the 100-W UV lamp (SXT-20-M, UVitec) was used as a light source. To prepare the suspension of the photocatalyst, 50 mg of Ga_2_O_3_ nanorod powders was mixed with 50 ml of RhB aqueous solution (2 × 10^−4^ M), which was continuously stirred at room temperature in the dark for 30 min. Then, the suspension was irradiated at different illumination times from 30 to 180 min. After that, the aliquot was separated from Ga_2_O_3_ nanorod powders by using a filter paper and then the absorbance of RhB solution was obtained using a UV-vis spectrophotometer (CARY 300 Bio, Varian).

## Results and Discussion

**Fig. 1:**
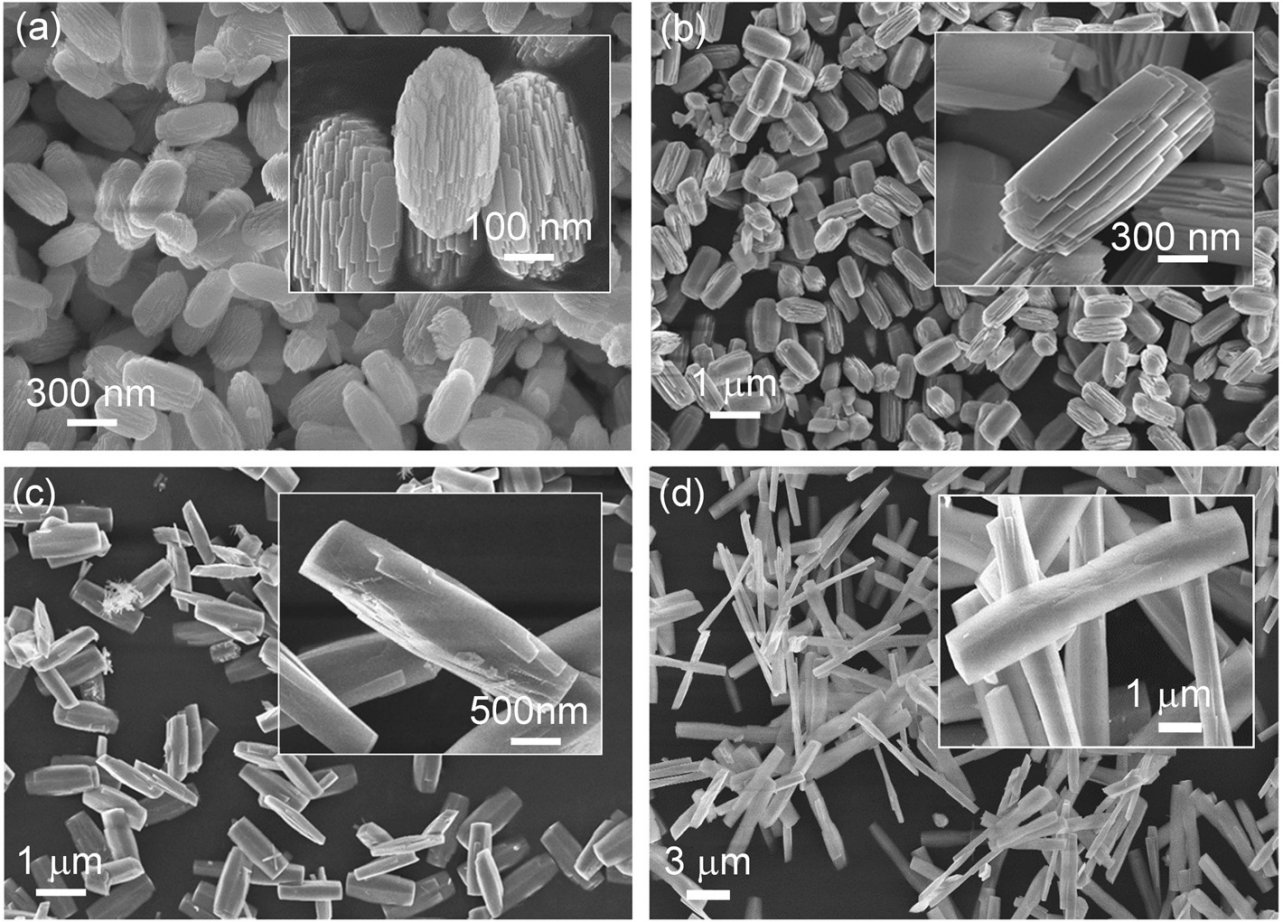
Effect of reaction temperature on the morphology of GaOOH nanostructures. FE-SEM images of the grown GaOOH nanostructures via hydrothermal method with different growth temperatures of **a** room temperature, **b** 50 °C, **c** 75 °C, and **d** 95 °C for 5 h

Figure 1 shows the FE-SEM images of GaOOH nanostructures synthesized via the hydrothermal method with different growth temperatures of (a) room temperature, (b) 50 °C, (c) 75 °C, and (d) 95 °C for 5 h. In general, the growth temperature plays an important role in controlling the size and shape of GaOOH nanostructures during the hydrothermal process. As shown in Fig. 1a, the cocoon-shaped GaOOH nanostructures were formed at room temperature and their size was approximately 100 nm. These cocoon-shaped structures were formed by multi-layers of small nanoplates. As the growth temperature was increased to 50 °C, the nanoplates merge together with the increase in thickness and width of each nanoplate due to Ostwald’s ripening as seen in Fig. 1b. Ostwald’s ripening continues further when the growth temperature is up to 75 °C and the multi-layered stacked structures were converted to a rod-shaped structure (Fig. 1c). With further increase of the growth solution temperature (95 °C), nanorods grow lengthwise and the width of the rods decreases as can be seen in Fig. 1d. The inset of Fig. 1d shows a single nanorod with lengths of a few micrometers and widths of approximately 1 μm. From the inset, we can also observe that the nanorod is ready to split into two rods of smaller size if the temperature of the growth solution is further increased.

**Fig. 2:**
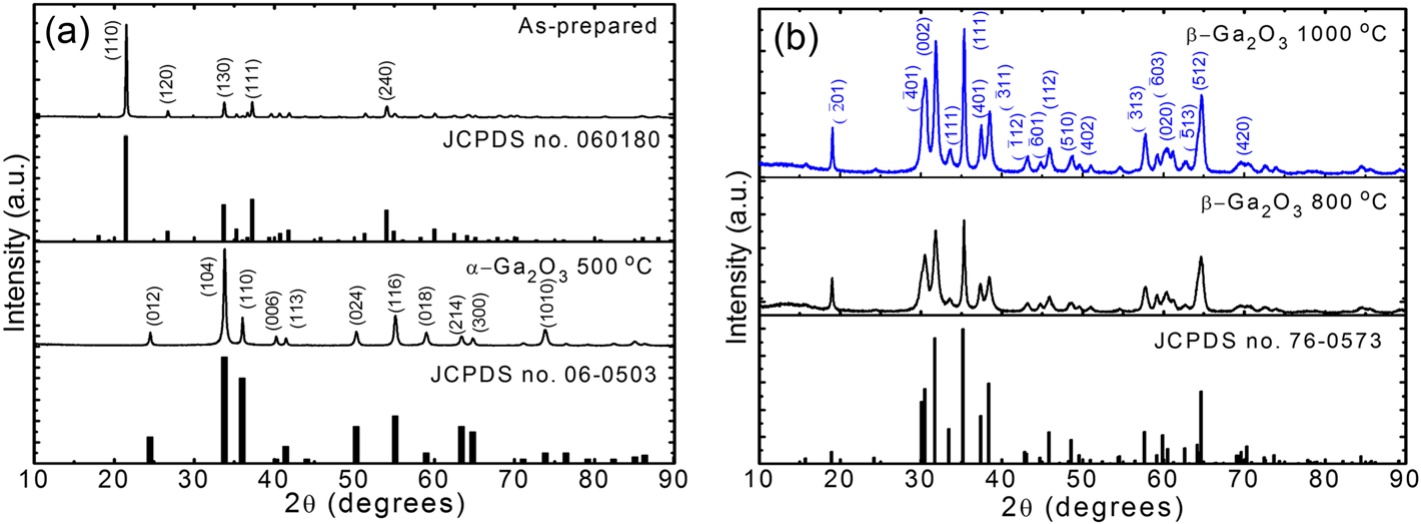
XRD of samples calcinated at different temperatures. 2*θ* scanned XRD patterns of **a** the as-prepared GaOOH nanorods and α-Ga_2_O_3_ calcined at 500 °C for 3 h and **b** the β-Ga_2_O_3_ calcined at 800 and 1000 °C for 3 h. Here, the GaOOH nanorods were hydrothermally grown at 95 °C

Figure 2 shows the 2*θ* scanned XRD patterns of (a) the as-prepared GaOOH nanorods and α-Ga_2_O_3_ calcined at 500 °C and (b) the β-Ga_2_O_3_ calcined at 800 and 1000 °C for 3 h. Here, the GaOOH nanorods were hydrothermally grown at 95 °C. As shown in Fig. 2a, the measured XRD peaks of the as-prepared GaOOH material well agreed with the crystallized orthorhombic structure (JCPDS no. 06-0180). The sharp dominant peak of the crystal planes (110), (130), (111), and (240) were observed at 2*θ* = 21.5, 33.7, 37.2, and 54.02°, respectively. For the calcined sample at 500 °C, it was observed that the as-prepared GaOOH nanorods were converted into α-Ga_2_O_3_ of the orthorhombic structure (JCPDS no. 06-0503) with dominant XRD peaks of (104) and (110) planes. When the calcination temperature was increased above 500 °C, as shown in Fig. 2b, the samples were changed into a monoclinic β-Ga_2_O_3_ (JCPDS no. 76-0573) without any impurity peaks. As the calcination temperature was increased to 1000 °C, the XRD patterns were clearly enhanced with sharp peaks owing to the improved crystallinity at relatively high calcination temperature. The sharp dominant peaks of the ($\overline{4}01$), (002), (111), and (512) planes were observed at 2*θ* = 30.4, 31.8, 35.3, and 64.71°, respectively.

**Fig. 3:**
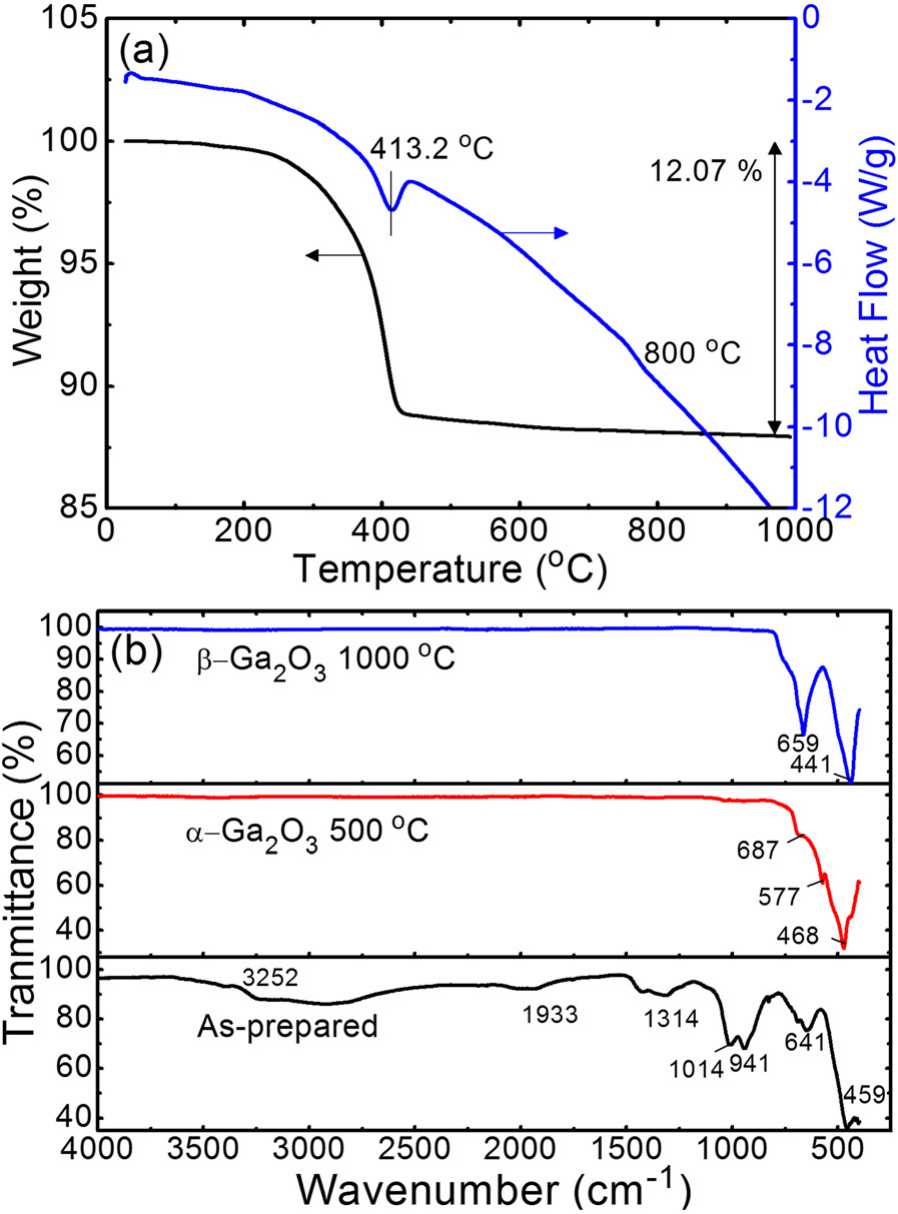
TGA-DSC curve and FT-IR spectra. **a** Measured TGA-DSC curves of the as-prepared GaOOH nanorods by heating from room temperature to 1000 °C with a fixed rate of 10 °C/min in a nitrogen atmosphere and **b** measured FT-IR spectra in the range of 4000 to 400 cm^−1^ for the as-prepared GaOOH, α-Ga_2_O_3_, and β-Ga_2_O_3_ nanorods

Figure 3a shows the measured TGA-DSC curves of the as-prepared GaOOH nanorods by heating from room temperature to 1000 °C with a fixed rate of 10 °C/min in a nitrogen atmosphere. In the TGA curve of GaOOH, the total weight loss was 12.07 % from 200 to 800 °C from the weight loss step process due to the physical removal of the absorbed water on the surface of GaOOH. For the endothermic peaks at 413.2 °C in the DSC curve, the phase transformation of GaOOH into α-Ga_2_O_3_ is well consistent with the measured XRD patterns of Fig. 2a. The weak endothermic peak was also observed at the temperature between 750 and 830 °C with a weight loss of 2 % for the conversion from α-Ga_2_O_3_ to β-Ga_2_O_3_ phase in the DSC curve. With further prolonged heating up to 1000 °C, there is no weight loss. Figure 3b shows the measured FT-IR spectra in the range of 4000 to 400 cm^−1^ for the as-prepared GaOOH, α-Ga_2_O_3_, and β-Ga_2_O_3_ nanorods. The as-prepared GaOOH exhibited two broad band peaks around 3252.17 and 1932.62 cm^−1^ which are assigned to the stretching vibration of H-O-H bands. Also, the stretching vibration bond of the O-H group is also observed around 1314 cm^−1^ owing to the absorption of water molecules. The constitutional bending vibration of Ga-OH bands was observed at 1014 and 941 cm^−1^. As the sample was calcined above 500 °C, the peaks disappeared in the range of 4000–700 cm^−1^ due to the dehydration of water molecules. For the α-Ga_2_O_3_ nanorods calcined at 500 °C, the weak stretching peaks of the Ga-O band were observed at 687, 577, and 468 cm^−1^, respectively. In contrast, the β-Ga_2_O_3_ nanorods calcined at 1000 °C clearly revealed the strong and narrow peaks at 659 and 441 cm^−1^, respectively. These FT-IR results well agreed with the trend of crystallinity for the Ga_2_O_3_ nanorods with different calcination temperatures from the XRD data (Fig. 2).

**Fig. 4:**
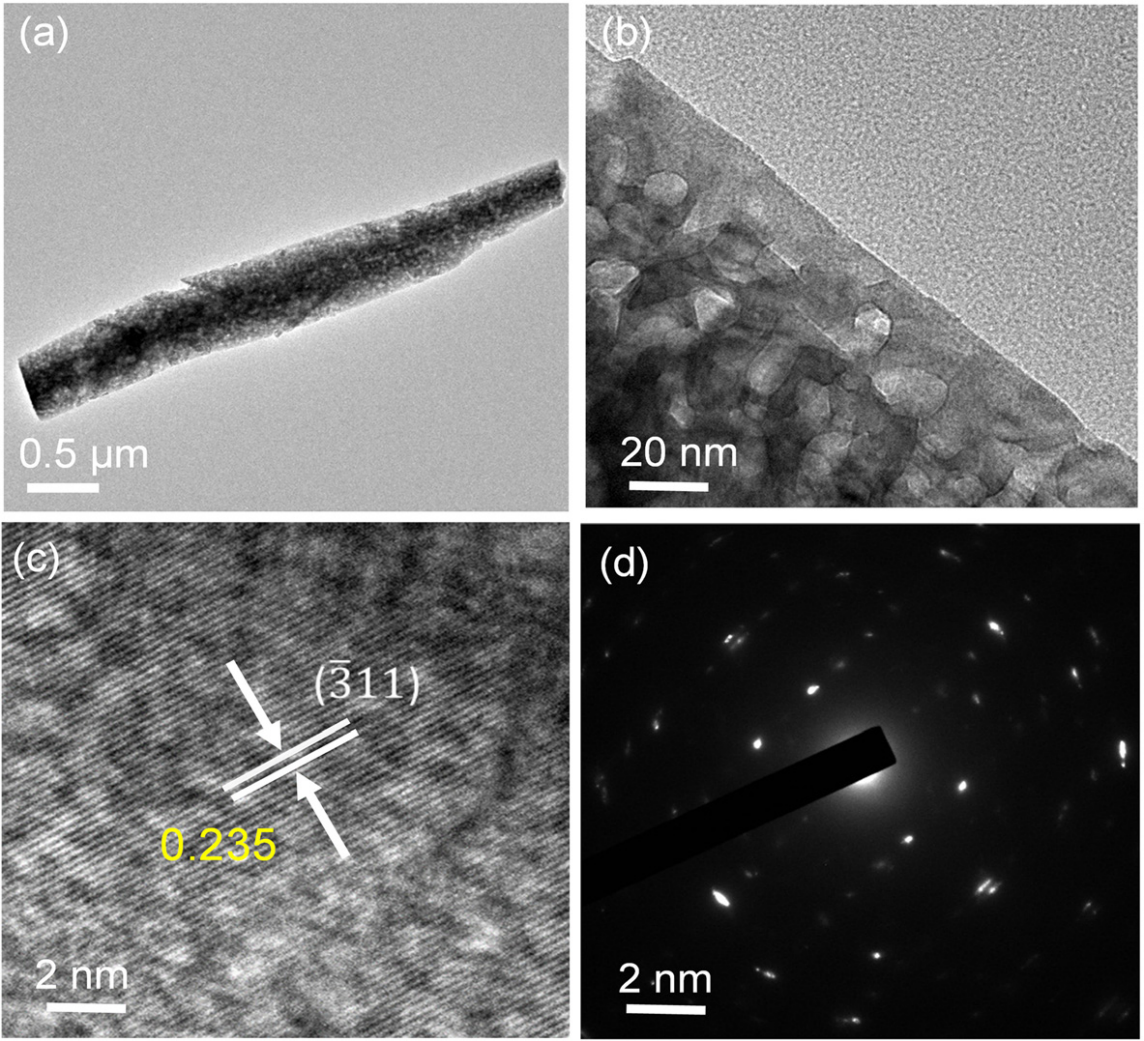
TEM images of β-Ga_2_O_3_ nanorods. **a**, **b** TEM images and **c** HRTEM image of the calcined β-Ga_2_O_3_ nanorods at 1000 °C. The SAED pattern of the corresponding sample is shown in **d**

Figure 4 shows the TEM images and selected area electron diffraction (SAED) patterns of β-Ga_2_O_3_ nanorods calcined at 1000 °C. As can be seen in the perspective view of the TEM image (Fig. 4a), the single β-Ga_2_O_3_ nanorod exhibited a length of ~3 μm and a diameter of ~280–400 nm with many nanoholes. The porous structure of β-Ga_2_O_3_ nanorods was also previously reported by Liu et al. and Prakasham et al. [28, 29]. This pore surface of β-Ga_2_O_2_ nanorods was formed during the decomposition since water molecules were eliminated, thus leading to the creation of a lot of vacancies. In Fig. 4b, the many holes had a size of ~20 nm, as clearly observed in the high-magnification view of the TEM image. From the high-resolution TEM (HRTEM) images in Fig. 4c, the line array of lattice fringes ($\overline{3}11$) was measured with a spacing of *d* = 0.235 nm, which is reasonable in comparison with the measured crystal plane of the corresponding XRD peak. From the SAED pattern of β-Ga_2_O_3_ nanorods, it was also supported that the GaOOH nanorods could be well converted into the β-Ga_2_O_3_ phase with good crystallinity by calcinating at 1000 °C.

**Fig. 5:**
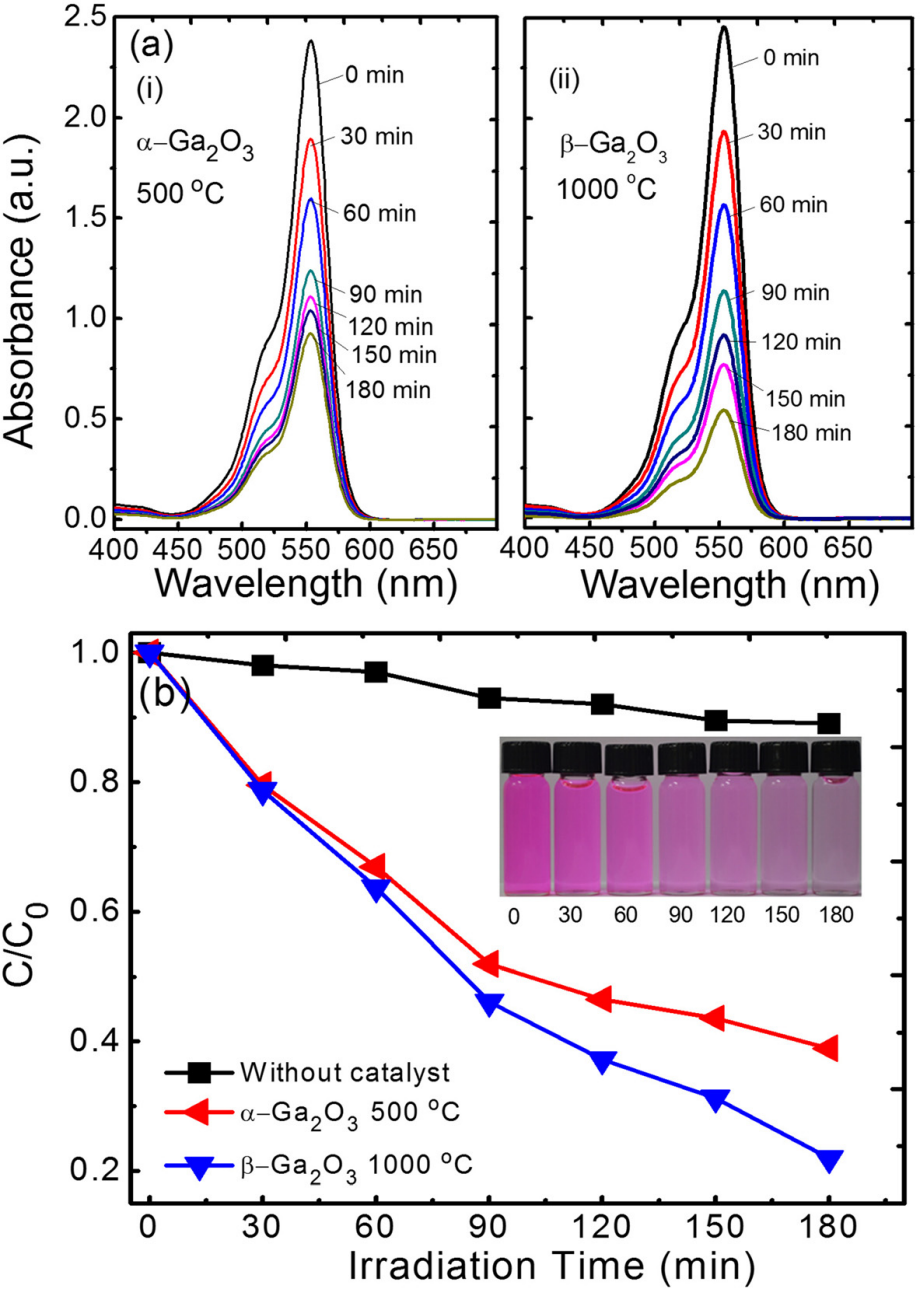
Photocatalytic activity of α-Ga_2_O_3_ and β-Ga_2_O_3_ under UV irradiation. Measured absorbance spectra of the RhB solution at different UV irradiation times in the presence of **a** (i) α-Ga_2_O_3_ and (ii) β-Ga_2_O_3_ nanorods. **b** Calculated constant reaction rate of the photodegradation of RhB solution (*C*/*C*_0_) as a function of UV irradiation time for α-Ga_2_O_3_ and β-Ga_2_O_3_ nanorods. The photographic images of the RhB solution with β-Ga_2_O_3_ nanorods at different UV irradiation times are also shown in the inset of **b**

To examine the photocatalytic activity of α-Ga_2_O_3_ and β-Ga_2_O_3_ nanorods, the photodegradation of RhB solution containing each sample was characterized. Figure 5 shows the measured absorbance spectra of the RhB solution at different UV irradiation times in the presence of (a) (i) α-Ga_2_O_3_ and (ii) β-Ga_2_O_3_ nanorods. From both the absorbance spectra, it is commonly observed that the absorption maximum of RhB occurred at 553 nm. As the UV irradiation time was increased from 0 to 60 min, the α-Ga_2_O_3_ and β-Ga_2_O_3_ nanorods exhibited a similar degradation of the absorption maximum. However, the absorbance peak of α-Ga_2_O_3_ nanorods was slowly reduced for longer UV irradiation time than 90 min. Meanwhile, the RhB solution shows the steady photodegradation up to 180 min of UV irradiation time by the photocatalyst of β-Ga_2_O_3_ nanorods. In general, the photocatalytic adsorption and desorption equilibrium are followed by a well-known Langmuir-Hinshelwood mechanism [30]. When the photon was absorbed into the photocatalyst, the photogenerated electron-hole separation contributed to the degradation of the RhB dye. Herein, this photocatalytic activity closely depends on the crystallinity and surface area [31–33]. The good crystallinity and porous surface of β-Ga_2_O_3_ nanorods, from the XRD and TEM analyses (Figs. 2 and 4), led to the stable photocatalytic property compared to the α-Ga_2_O_3_ nanorods. Figure 5b shows the calculated constant reaction rate of the photodegradation of RhB solution (*C*/*C*_0_) as a function of UV irradiation time for α-Ga_2_O_3_ and β-Ga_2_O_3_ nanorods. Here, *C*_0_ is the initial concentration of the RhB dye and *C* is the concentration of the UV-irradiated RhB solution. For comparison, the self-degradation rate of RhB solution was also shown by obtaining the *C*/*C*_0_ without photocatalyst. When the UV irradiation time was increased, the β-Ga_2_O_3_ nanorods provided a more efficient constant reaction rate. At 180 min of UV irradiation time, the photodegradation efficiency reached to 62 and 79 % for the α-Ga_2_O_3_ and β-Ga_2_O_3_ nanorods, respectively. The inset of Fig. 5b shows the photographic images of the RhB solution with β-Ga_2_O_3_ nanorods at different UV irradiation times. As the UV irradiation time was increased, it is clearly observed that the initial pink color of the RhB solution gradually disappeared, exhibiting a colorless solution. These fabrication methods and characterizations suggest that hydrothermally synthesized β-Ga_2_O_3_ nanorods can be expected to be a promising alternative for photocatalysts in the environmental remediation process.

## Conclusions

The GaOOH nanorods with a length of ~1 μm and a width of ~300–400 nm were successfully prepared by a simple hydrothermal synthesis at 95 °C of growth temperature. Then, the as-prepared GaOOH nanorods were calcined at different temperatures of 500–1000 °C for converting into single crystalline α-Ga_2_O_3_ and β-Ga_2_O_3_ nanorods, and their crystal structures were confirmed by the XRD analysis. Also, the dehydration processes were studied by thermal analysis with consideration of the phase changes of the as-prepared GaOOH precursors. At 1000 °C of calcination temperature, the β-Ga_2_O_3_ nanorods with good crystallinity and porous surface were formed by the removal of water molecules during the dehydration. Additionally, these β-Ga_2_O_3_ nanorods provided a relatively stable and high photocatalytic activity, compared with the α-Ga_2_O_3_ nanorods. Under UV irradiation for 180 min, the β-Ga_2_O_3_ nanorods exhibited a relatively high photodegradation efficiency of 79 % compared to the α-Ga_2_O_3_ nanorods (62 %). This fabrication process and analysis can be useful to produce a good inorganic semiconductor nanomaterial-based photocatalyst.
